# Defining the clinical characteristics of mammary analog secretory carcinoma of the salivary gland: Analysis of the National Cancer Database

**DOI:** 10.1177/20503121231200103

**Published:** 2023-09-25

**Authors:** Akshilkumar Patel, Brandon LaBarge, Tonya S King, Sandeep Pradhan, Joshua Warrick, Neerav Goyal

**Affiliations:** 1 The Pennsylvania State University College of Medicine, Hershey, PA, USA; 2 Department of Otolaryngology—Head and Neck Surgery, Penn State Health Milton S. Hershey Medical Center, Hershey, PA, USA; 3 Department of Public Health Sciences, The Pennsylvania State University College of Medicine, Hershey, PA, USA; 4 Department of Pathology and Laboratory Medicine, Penn State Health Milton S. Hershey Medical Center, Hershey, PA, USA

**Keywords:** Mammary analog secretory carcinoma, salivary gland neoplasms, head and neck neoplasms, mouth neoplasms

## Abstract

**Objectives:**

Mammary analog secretory carcinoma (MASC) is a classification of salivary gland tumors, recently included within the term secretory carcinoma. Previous descriptions of this diagnosis have largely consisted of case reports and case series with few studies investigating its clinical characteristics as compared to non-MASC tumors. Our objective was to use a large patient database to compare the clinical characteristics of mammary analog secretory carcinoma vs. non-mammary analog secretory carcinoma salivary gland tumors.

**Methods:**

The National Cancer Database was queried between September and October 2022 for histological diagnosis of mammary analog secretory carcinoma and non-MASC salivary tumors. Patients diagnosed with mammary analog secretory carcinoma and non-mammary analog secretory carcinoma salivary tumors between the period of 2004 through 2019 were included in this analysis. Various demographic and clinical variables were abstracted from the database and compared using Wilcoxon rank sum and chi-square tests. Survival was compared between cohorts using Cox proportional hazards regression.

**Results:**

Overall, compared to non-mammary analog secretory carcinoma diagnoses (*n* = 47668), mammary analog secretory carcinoma tumors (*n* = 384) affected younger individuals, displayed favorable pathologic staging and tumor grade, and were less likely to invade surrounding tissues. Patients with mammary analog secretory carcinoma tumors also received treatment more quickly following diagnosis compared to patients with non-mammary analog secretory carcinoma tumors. The risk of death was 4.3 times greater for non-mammary analog secretory carcinoma diagnoses when adjusted for patient variables (hazard ratio = 4.3, 95% confidence interval [2.37–7.71], *p* < 0.001).

**Conclusions:**

Clinically, mammary analog secretory carcinoma salivary tumors have a more indolent course compared to other salivary cancers. Additional studies are needed to determine the natural history of this tumor type.

## Introduction

The 2017 World Health Organization (WHO) classification of head and neck tumors characterized the diverse group of salivary gland carcinomas by demographic, clinical, and histologic features.^1^ This 4th edition included mammary analog secretory carcinoma (MASC) as an entity distinct from acinic cell carcinoma.^2^ In the recently updated 5th edition of WHO classification of head and neck salivary gland tumors, secretory carcinomas harboring an *ETV6*-related mutation were grouped under the umbrella term secretory carcinoma (SC).^3^

Current literature describing MASC of salivary glands predominantly features case reports and case series, with a focus on cytopathologic features. MASC of salivary glands was first described as a distinct tumor syndrome in 2010 by Skalova *et al*. (2010).^4^ Genetically, MASC is characterized by a t(12;15)(p13;q25) *ETV6-NTRK3* gene translocation that is also shared by secretory carcinoma of the breast, collectively termed SC.^2,5^ Before being distinguished as a separate entity, MASC tumors were erroneously miscategorized as other salivary gland neoplasms, such as acinic cell carcinoma and adenocarcinoma. In addition to the t(12;15) *ETV6-NTRK3* translocation, several other mutations have been implicated in the pathogenesis of MASC, including novel *VIM-RET, EGFR-SEPT14*, and *ETV6-MAML3* gene fusions, helping to further distinguish MASC from its mimics.^6–8^ Together, genetic, immunohistochemical, and histopathologic findings are factors that distinguish MASC as a distinct entity of SC.

Clinically, the literature describes cases of MASC invading various body tissues, including the maxillary and ethmoid sinuses, buccal mucosa, the palate, thyroid gland, lung, liver, and vulva, and affects children and adults alike.^9–16^ Meaningful synthesis of large-scale clinical data describing MASC, however, is lacking. Although Anderson *et al*. (2019) reported on MASC patients identified through the Surveillance, Epidemiology, and End Results database, only 55 patients were identified and analyzed.^17^ Larger cohorts of patients have yet to be studied; thus, many questions remain regarding demographics and outcomes for patients with these tumors, including the comparison to non-MASC malignant salivary tumors. The goal of this study was to analyze a comprehensive cancer database to compare clinical trends in MASC vs. non-MASC salivary tumors.

## Materials and methods

This study is a retrospective cohort study. National Cancer Database (NCDB) data were accessed between September and October 2022, and histological diagnoses of MASC and non-MASC salivary malignancies were collected over the period of 2004 through 2019.^18^ Subsequent analyses compared MASC diagnoses to a select cohort of non-MASC diagnoses, namely squamous cell carcinoma, adenocarcinoma, adenoid cystic carcinoma, mucoepidermoid carcinoma, lobular carcinoma, and acinar cell carcinoma. Finally, individual comparisons between MASC and each of the six individual non-MASC diagnoses included in the cohort were also performed. Data abstracted for both diagnosis categories include patient demographics (age, sex, race, income, and education), year of diagnosis, primary tumor site, grade, stage, size, nodal status, tumor extension, lymphovascular invasion, surgical treatment and approach, margin status, radiation therapy, chemotherapy, and patient mortality. Inclusion criteria included all adults (aged ⩾18) with a listed diagnosis of MASC and non-MASC salivary cancer, and exclusion criteria included patients with diagnoses of non-salivary gland cancer.

### Statistical analysis

Demographic and clinical characteristics were compared between the MASC and non-MASC tumor cohorts using the Wilcoxon rank sum test for continuous measures and the chi-square test for categorical measures.^19^ A Kaplan–Meier curve was generated to compare survival between cohorts, and Cox proportional hazards regression models were fit both with and without adjustment for important baseline characteristics. All statistical analyses were performed using SAS version 9.4 (SAS Institute Inc, Cary, NC), and statistical significance was defined as *p* < 0.05.

## Results

During the study period, a total of 384 cases of MASC and 47,668 cases of non-MASC tumors were collected from the NCDB, with only one MASC case (identified in 2006) identified prior to 2010. Table 1 demonstrates the demographic characteristics of MASC and non-MASC patients. On average, patients with MASC salivary tumors were younger at the time of initial diagnosis compared to patients with non-MASC salivary tumors (53.4 years vs. 63.6 years, *p* < 0.001). A lower proportion of MASC cases were male compared to non-MASC cases (50.8% vs. 57.1%, *p* = 0.013). There was a significant difference between cohorts with respect to the distribution of race (*p* < 0.001) and Spanish-Hispanic ethnic origin (*p* = 0.004). Although there was white race and non-Spanish, non-Hispanic ethnic origin predominance among both populations, the non-MASC cohort had more white patients (84.3% vs. 77.6%), while the MASC cohort had slightly more non-Spanish, non-Hispanic patients (90.9% vs. 89.9%).

**Table 1.** Demographic characteristics of MASC vs. non-MASC tumors.

Demographic characteristic	Histological type		*p*-Value
	MASC (*N* = 384)	Non-MASC (*N* = 47,668)	
Age at diagnosis, mean (SD)	53.4 (17.17)	63.6 (16.35)	<0.001^a^
Sex: male, *n* (%)	195 (50.8%)	27,227 (57.1%)	0.0125^b^
Race, *n* (%)			<0.001^b^
White	298 (77.6%)	40,065 (84.3%)	
Black	39 (10.2%)	4669 (9.8%)	
Asian/Pacific Islander	35 (9.1%)	1674 (3.5%)	
Other/unknown	12 (3.1%)	1131 (2.4%)	
Spanish Hispanic origin, *n* (%)			0.0040^b^
Non-Spanish, non-Hispanic	349 (90.9%)	42,863 (89.9%)	
Spanish, Hispanic	29 (7.6%)	2588 (5.4%)	
Unknown	6 (1.6%)	2217 (4.7%)	

a Wilcoxon Rank Sum *p*-value.

b Fisher’s exact *p*-value.

Table 2 describes the clinical characteristics of MASC and non-MASC tumors. There was a significant difference between cohorts with respect to the primary tumor site (*p* = 0.001), with MASC tumor patients more likely to have parotid gland involvement within the primary tumor site (84.9% vs. 80.5%). MASC patients also exhibited lower rates of lymphovascular invasion (7.8% vs. 19.4%, *p* < 0.001) and were more likely to demonstrate negative lymph node status at diagnosis where examined (82.7% vs. 61.3%, *p* < 0.001).

**Table 2.** Clinical characteristics of MASC vs. non-MASC tumors.

Clinical characteristic	Histological type		*p*-Value
	MASC (*N* = 384)	Non-MASC (*N* = 47,668)	
Lymphovascular invasion: not present, *n* (%)	294 (92.2%)	16,954 (76.2%)	<0.001^a^
Primary site, *n* (%)			0.0014^a^
Parotid gland	326 (84.9%)	38,393 (80.5%)	
Submandibular gland	31 (8.1%)	5898 (12.4%)	
Sublingual gland	0 (0.0%)	528 (1.1%)	
Overlapping lesion of major salivary glands	3 (0.8%)	91 (0.2%)	
Major salivary gland, not otherwise specified	24 (6.3%)	2758 (5.8%)	
Regional lymph nodes positive, *n* (%)			<0.001^a^
All nodes examined are negative	225 (82.7%)	19,091 (61.3%)	
Any positive nodes	47 (17.3%).	12,053 (38.7%)	

a Fisher’s exact *p*-value.

Table 3 presents the treatment characteristics of patients in the MASC and non-MASC tumor cohorts. Overall, MASC patients were able to receive treatment in fewer days after diagnosis than non-MASC patients (18.0 days vs. 21.4 days, *p* < 0.001), including fewer days to the first surgical procedure compared to non-MASC patients (16.8 days vs. 19.8 days, *p* < 0.001). MASC patients were also more likely to undergo a surgical treatment compared to non-MASC patients (96.9% vs. 90.1%, *p* < 0.001), with a significant difference between cohorts with respect to surgical margin status (*p* < 0.001). MASC tumors showed a higher frequency of cases with negative surgical margins during surgical resection (85.6% vs. 66.4%). Fewer MASC cases required treatment with radiation therapy (30.6% vs. 56.8%, *p* < 0.001) and chemotherapy (4.2% vs. 13.3%, *p* < 0.001).

**Table 3.** Treatment demographics of MASC vs. non-MASC tumors.

Treatment demographic	Histological type		*p*-Value
	MASC (*N* = 384)	Non-MASC (*N* = 47,668)	
Treatment started, days from diagnosis: mean (SD)	18.0 (38.64)	21.4 (38.84)	<0.001^a^
First surgical procedure, days from diagnosis: mean (SD)	16.8 (33.30)	19.8 (39.43)	0.0002^a^
Surgery, *n* (%)			<0.001^b^
No	12 (3.1%)	4702 (9.9%)	
Yes	372 (96.9%)	40,230 (90.1%)	
Surgical margin status, *n* (%)			<0.001^b^
No residual tumor, all margins negative	310 (85.6%)	25,181 (66.4%)	
Residual tumor, not otherwise specified	19 (5.2%)	4983 (13.1%)	
Microscopic residual tumor	33 (9.1%)	7282 (19.2%)	
Macroscopic residual tumor	0 (0.0%)	499 (1.3%)	
Radiation therapy, *n* (%)			<0.001^b^
No	261 (69.4%)	19,897 (43.2%)	
Yes	123 (30.6%)	27,771 (56.8%)	
Chemotherapy, *n* (%)			0.0005^b^
No	368 (95.8%)	41,328 (86.7%)	
Yes	16 (4.2%)	6334 (13.3%)	

a Wilcoxon rank sum *p*-value.

b Fisher’s exact *p*-value.

Table 4 shows the difference in clinical grades of patients diagnosed with MASC and non-MASC tumors (*p* < 0.001). MASC patients were found to exhibit significantly higher rates of well- to moderately-differentiated tumors histologically compared to non-MASC patients (93.9% vs. 51.6%), among those with known grades. Table 4 also highlights the American Joint Committee on Cancer (AJCC) pathologic staging of MASC vs. non-MASC tumors. There was a significant difference between MASC and non-MASC cases with respect to AJCC pathologic grade (*p* < 0.001), with MASC cases being more likely to be diagnosed at stage 1 (46.9% vs. 26.7%).

**Table 4.** Clinical grades and AJCC pathologic staging of MASC vs. non-MASC tumors.

Pathology characteristic	Histological type		*p*-Value
	MASC (*N* = 384)	Non-MASC (*N* = 47,668)	
Grade, *n* (%)			<0.001^a^
Well-differentiated, moderately-differentiated	107 (93.9%)	14,084 (51.6%)	
Poorly-differentiated	4 (3.5%)	10,665 (39.0%)	
Undifferentiated	3 (2.6%)	2592 (9.5%)	
AJCC Pathologic Stage Group, *n* (%)			<0.001^a^
pStage I	84 (46.9%)	7474 (26.7%)	
pStage II	48 (26.8%)	5951 (21.2%)	
pStage III	29 (16.2%)	5597 (20.0%)	
pStage IV	0 (0.0%)	1 (0.0%)	
pStage IVA	14 (7.8%)	237 (0.8%)	
pStage IVB	1 (0.6%)	7029 (25.1%)	
pStage IVC	3 (1.7%)	722 (2.6%)	

a Fisher’s exact *p*-value.

Overall, there was a significant difference in the survival of MASC vs. non-MASC patients according to the log-rank test (unadjusted *p* < 0.001), with 17 out of 308 MASC patients with survival data experiencing death compared to 18,529 out of 44,217 non-MASC patients. With adjustment for age, insurance type, clinical stage of tumor, pathological stage of tumor, and regional lymph node involvement, the risk of death was 4.3 times greater for non-MASC vs. MASC patients (hazard ratio = 4.3, 95% confidence interval [2.37–7.71], *p* < 0.001).

The relationship between MASC cases and a select group of non-MASC cases (squamous cell carcinoma, adenocarcinoma, adenoid cystic carcinoma, mucoepidermoid carcinoma, lobular carcinoma, and acinar cell carcinoma) was also investigated (Supplemental Tables 1–7). Overall, these additional comparisons recapitulate the findings of MASC tumors affecting younger, male patients, leading to a less severe clinical course and better treatment-based outcomes. With adjustment for age, insurance type, clinical stage of tumor, pathological stage of tumor, and regional lymph node involvement, the risk of death was 4.1 times greater for the select group of non-MASC vs. MASC patients (HR = 4.1, 95% CI [2.30–7.48], *p* < 0.001).

## Discussion

MASC is a recently recognized histological type of salivary gland tumor, and despite previous attempts to characterize its clinical properties, a comprehensive analysis comparing MASC to non-MASC salivary gland tumors has not yet been reported in the literature. In comparison to non-MASC salivary tumors, our analysis demonstrates MASC tumors are more likely to affect younger patients, be well- to moderately-differentiated, have decreased rates of lymphovascular invasion, and have less nodal disease. In comparison to the findings published by Anderson *et al*. (2019), our results support the findings of MASC tumors affecting younger, male patients with the majority of cases confined to the parotid gland without lymph node involvement, representing low AJCC staging, and demonstrating favorable histological grading.^17^ However, in contrast to Anderson *et al*. (2019), our study demonstrates the increased mortality of non-MASC tumor cases compared to MASC tumors. From an oncologic treatment perspective, MASC tumors exhibit less aggressive staging, less likelihood of distant metastasis with a higher proportion of isolation to the gland, increased rates of negative lymph nodal disease, decreased lymphovascular invasion, and fewer cases requiring radiation-based treatment. Furthermore, patients with a MASC diagnosis received treatment, including the first surgical procedure, in less time following diagnosis compared to patients with a non-MASC diagnosis. The findings suggest that MASC salivary tumors are less aggressive than non-MASC malignant salivary tumors. Our data are consistent with prior reports and add additional measures not previously studied. This conclusion is further supported by non-MASC diagnoses demonstrating a higher risk of death than MASC diagnoses.

The literature contains previous reports differentiating MASC from other salivary gland tumors. In a retrospective analysis by Alves *et al*. (2021), post-diagnostic review revealed several cases of SC as previously diagnosed with acinic cell carcinoma.^20^ Histologically, *MUC4* mutations represent sensitive and specific markers for distinguishing MASC from other forms of salivary tumors, with high positive and negative predictive values.^21^ MASC tumors have also been described in non-salivary locations, including the lip, soft palate, and mandibular vestibule.^22^ Pathologic parameters of MASC tumor cells, including prevailing architecture, pleomorphism, tumor necrosis, perineural invasion, lymphovascular invasion, and mitotic count and/or Ki-67 labeling index, have also been proposed to differentiate cases into a three-tiered grading system.^23^ Currently, the majority of salivary SCs are diagnosed via histopathology, although case reports describing the radiographic properties of these rare tumors may contribute to diagnosing without necessitating biopsy.^24,25^ In a pediatric patient, magnetic resonance imaging (MRI) of a parotid-based MASC tumor demonstrated a complex cystic and solid mass, with the cystic components exhibiting hyperintensity on T1- and T2-weighted imaging, and the solid components exhibiting hypointensity on T2-weighted imaging.^24^ This MRI finding is similarly present in adult cases of MASC, which also demonstrates MASC tumors present as predominantly cystic tumors with hyperintensity on T1-weighted images and variable signal intensity on T2-weighted images.^25^

A systematic review of salivary gland SCs reported 403 cases of MASC tumors in the current literature after histopathological reclassification of cases previously diagnosed as other salivary gland tumors.^26^ Their findings recapitulate those described in the present study, namely, MASC diagnoses more commonly affected males, affected the parotid gland, and were commonly treated surgically, with isolated cases of tumor recurrence, metastasis, and death.^26^ Furthermore, in a recent study by Silver *et al.* (2022), among a total of 657 cases, 46 (7.0%) initially presented with either locoregional or distant metastasis, while 14 (2.1%) resulted in deaths.^27^ Our data build upon this knowledge through a direct comparison between MASC and non-MASC diagnosis among a large patient cohort and furthers knowledge regarding long-term outcomes for MASC patients. As a systematic review, the study by Alves *et al*. (2021) is inherently limited as the majority of MASC cases in the current literature, including 74 out of a total 119 studies yielded by the systematic review, are case reports prone to selection and publication biases, which are not as evident in data extracted from an extensive, national patient database.

The present study highlights clinical distinctions among patients with both MASC and non-MASC salivary tumors. The *ETV6-NTRK3* gene translocation provides an additional source of targeted therapy. Previously, the TRK protein inhibitor Larotrectinib was used to treat a pediatric patient diagnosed with an *ETV6-NTRK3* fusion-positive secretory breast carcinoma.^28^ Recent data from multiple clinical trials using Larotrectinib further bolster its utility in the treatment of TRK fusion-positive cancer, with 121 (79.1%) of 153 patients demonstrating an objective response, with 24 (15.7%) having a complete response.^29^ Furthermore, a variety of tumor types harbor the *ETV6-NTRK3* fusion protein, thus providing additional targets for future TRK inhibition therapy.^30^

Our study is not without limitations in both data collection and review. As a retrospective analysis of a preexisting patient database, we are inherently limited by the data that are both reported and lacking within the database, in addition to inconsistent reporting across all patients. Therefore, we are unable to analyze individual patients’ genetic or histological records to confirm the accuracy of their diagnosis and must rely on the good faith of accurate reporting by clinicians to the National Cancer Database. In addition, our analysis includes data ranging from 2004 through 2019, despite MASC being classified as a distinct malignancy in 2010. Therefore, MASC tumors may have been misclassified as non-MASC diagnoses prior to 2010 and included in our analysis as non-MASC cancers. Our motivation for including data ranging from 2004 to 2019 was to maximize the number of patient diagnoses (of both MASC and non-MASC tumors) included in the analysis. Given its status as a rare disease, mortality data are available only for a limited number of MASC patients. Finally, power analysis for sample size calculation was not performed for this study; all MASC and non-MASC cases available within the 2004–2019 timeline were included in the study.

Due to its rare incidence and recent disease classification, the clinical features and natural history of MASC are still under investigation. Although our analysis of a comprehensive cancer database reveals demographic, clinical, and treatment differences in MASC vs. non-MASC salivary tumors, additional studies are needed to fully characterize this new diagnosis. We hope that as more data continue to become available, more effective therapeutic guidelines can be established for patients with this diagnosis.

## Conclusions

Overall, the findings suggest that MASC salivary tumors are less aggressive than non-MASC malignant salivary tumors. Our data are consistent with prior reports and add additional measures not previously studied. MASC tumors were found to affect younger, male patients with the majority of cases isolated to the parotid gland, representing low AJCC staging, and demonstrating favorable histology. Furthermore, patients with MASC tumor diagnoses received treatment quicker than patients with non-MASC tumors, in addition to experiencing decreased mortality compared to patients with a non-MASC salivary tumor diagnosis. Clinicians must become aware of the unique characteristics of MASC tumors in how they can be differentiated from non-MASC tumors, thus helping guide patient care and management.

## Supplemental Material

sj-docx-1-smo-10.1177_20503121231200103 – Supplemental material for Defining the clinical characteristics of mammary analog secretory carcinoma of the salivary gland: Analysis of the National Cancer Database Click here for additional data file. Supplemental material, sj-docx-1-smo-10.1177_20503121231200103 for Defining the clinical characteristics of mammary analog secretory carcinoma of the salivary gland: Analysis of the National Cancer Database by Akshilkumar Patel, Brandon LaBarge, Tonya S King, Sandeep Pradhan, Joshua Warrick and Neerav Goyal in SAGE Open Medicine
