# Supplementary material for: Defining the clinical characteristics of mammary analog secretory carcinoma of the salivary gland: Analysis of the National Cancer Database
Source: SAGE Open Med. 2023 Sep 25;11:20503121231200103. doi: 10.1177/20503121231200103 (PMC10521276; doi:10.1177/20503121231200103)
Supplement: sj-docx-1-smo-10.1177_20503121231200103 – Supplemental material for Defining the clinical characteristics of mammary analog secretory carcinoma of the salivary gland: Analysis of the National Cancer Database [file sj-docx-1-smo-10.1177_20503121231200103.docx]

Supplemental Table 1: Demographic, clinical, treatment, clinical grade, and AJCC pathologic staging characteristics of MASC vs. Select Non-MASC tumors.

|  | **Histological Type** | |  |
| --- | --- | --- | --- |
|  | **MASC (N=384)** | **Non-MASC (N=31400)** | **P-value** |
| **Demographic Characteristics** | | | |
| Age at Diagnosis, Mean (SD) | 53.4 (17.17) | 61.9 (17.00) | <0.001^1^ |
| Sex: Male, n (%) | 195 (50.8%) | 16913 (53.9%) | 0.2286^2^ |
|  | | | |
| **Race, n (%)** |  |  | <0.001^2^ |
| White | 298 (77.6%) | 26128 (83.4%) |  |
| Black | 39 (10.2%) | 3280 (10.5%) |  |
| Asian/Pacific Islander | 35 (9.1%) | 1107 (3.5%) |  |
| Other/Unknown | 12 (3.1%) | 804 (2.6%) |  |
|  | | | |
| **Spanish Hispanic Origin, n (%)** |  |  | <0.001^2^ |
| Non-Spanish, non-Hispanic | 349 (90.9%) | 28040 (89.3%) |  |
| Spanish, Hispanic | 29 (7.6%) | 1813 (5.8%) |  |
| Unknown | 6 (1.6%) | 1547 (4.9%) |  |
|  | | | |
| **Clinical Characteristics** | | | |
| Lympho-Vascular Invasion: Not present, n (%) | 294 (92.2%) | 11852 (80.6%) | <0.001^2^ |
|  | | | |
| **Primary Site, n (%)** |  |  | <0.001^2^ |
| Parotid gland | 326 (84.9%) | 25106 (80.0%) |  |
| Submandibular gland | 31 (8.1%) | 4126 (13.1%) |  |
| Sublingual gland | 0 (0.0%) | 448 (1.4%) |  |
| Overlapping lesion of major salivary glands | 3 (0.8%) | 58 (0.2%) |  |
| Major salivary gland, not otherwise specified | 24 (6.3%) | 1662 (5.3%) |  |
|  | | | |
| **Regional Lymph Nodes Positive, n (%)** |  |  | <0.001^2^ |
| All nodes examined are negative | 225 (82.7%) | 13595 (65.5%) |  |
| Any positive nodes | 47 (17.3%) | 7163 (34.5%) |  |
|  | | | |
| **Treatment Characteristics** | | | |
| Treatment Started, Days from Diagnosis: Mean (SD) | 18.0 (38.64) | 20.6 (37.29) | <0.001^1^ |
| First Surgical Procedure, Days from Diagnosis: Mean (SD) | 16.8 (33.30) | 19.1 (37.60) | <0.001^1^ |
|  | | | |
| **Surgery, n (%)** |  |  | <0.001^2^ |
| No | 12 (3.1%) | 4431 (14.1%) |  |
| Yes | 372 (96.9%) | 26969 (85.9%) |  |
|  | | | |
| **Surgical Margin Status, n (%)** |  |  | <0.001^2^ |
| No residual tumor, all margins negative | 310 (85.6%) | 17153 (66.9%) |  |
| Residual tumor, not otherwise specified | 19 (5.2%) | 3272 (12.8%) |  |
| Microscopic residual tumor | 33 (9.1%) | 4894 (19.1%) |  |
| Macroscopic residual tumor | 0 (0.0%) | 316 (1.2%) |  |
|  | | | |
| **Radiation Therapy, n (%)** |  |  | <0.001^2^ |
| No | 261 (69.4%) | 13543 (44.6%) |  |
| Yes | 123 (30.6%) | 16807 (55.4%) |  |
|  | | | |
| **Chemotherapy, n (%)** |  |  | 0.0095^2^ |
| No | 368 (95.8%) | 27831 (88.6%) |  |
| Yes | 16 (4.2%) | 3569 (13.3%) |  |
|  | | | |
| **Clinical Grades and AJCC Pathologic Staging** | | | |
| **Grade, n (%)** |  |  | <0.001^2^ |
| Well differentiated, moderately differentiated | 107 (93.9%) | 11212 (59.7%) |  |
| Poorly differentiated | 4 (3.5%) | 6361 (33.9%) |  |
| Undifferentiated | 3 (2.6%) | 1194 (6.4%) |  |
|  | | | |
| **AJCC Pathologic Stage Group, n (%)** |  |  | <0.001^2^ |
| pStage I | 84 (46.9%) | 5848 (30.5%) |  |
| pStage II | 48 (26.8%) | 4083 (21.3%) |  |
| pStage III | 29 (16.2%) | 3738 (19.5%) |  |
| pStage IV | 0 (0.0%) | 1 (0.0%) |  |
| pStage IVA | 14 (7.8%) | 143 (0.7%) |  |
| pStage IVB | 1 (0.6%) | 4279 (22.3%) |  |
| pStage IVC | 3 (1.7%) | 459 (2.4%) |  |
| ^1^Wilcoxon Rank Sum p-value; ^2^Fisher Exact p-value | | | |

Supplemental Table 2: Demographic, clinical, treatment, clinical grade, and AJCC pathologic staging characteristics of MASC vs. Squamous Cell Carcinoma (SCC).

|  | **Histological Type** | |  |
| --- | --- | --- | --- |
|  | **MASC (N=384)** | **SCC (N=7276)** | **P-value** |
| **Demographic Characteristics** | | | |
| Age at Diagnosis, Mean (SD) | 53.4 (17.17) | 73.3 (12.22) | <0.001^1^ |
| Sex: Male, n (%) | 195 (50.8%) | 5790 (79.6%) | <0.001^2^ |
|  | | | |
| **Race, n (%)** |  |  | <0.001^2^ |
| White | 298 (77.6%) | 6781 (93.4%) |  |
| Black | 39 (10.2%) | 276 (3.8%) |  |
| Asian/Pacific Islander | 35 (9.1%) | 96 (1.3%) |  |
| Other/Unknown | 12 (3.1%) | 110 (1.5%) |  |
|  | | | |
| **Spanish Hispanic Origin, n (%)** |  |  | <0.001^2^ |
| Non-Spanish, non-Hispanic | 349 (90.9%) | 6690 (91.9%) |  |
| Spanish, Hispanic | 29 (7.6%) | 226 (3.1%) |  |
| Unknown | 6 (1.6%) | 360 (4.9%) |  |
|  | | | |
| **Clinical Characteristics** | | | |
| Lympho-Vascular Invasion: Not present, n (%) | 294 (92.2%) | 1882 (72.6%) | <0.001^2^ |
|  | | | |
| **Primary Site, n (%)** |  |  | <0.001^2^ |
| Parotid gland | 326 (84.9%) | 6286 (86.4%) |  |
| Submandibular gland | 31 (8.1%) | 773 (10.6%) |  |
| Sublingual gland | 0 (0.0%) | 18 (0.2%) |  |
| Overlapping lesion of major salivary glands | 3 (0.8%) | 9 (0.1%) |  |
| Major salivary gland, not otherwise specified | 24 (6.3%) | 190 (2.6%) |  |
|  | | | |
| **Regional Lymph Nodes Positive, n (%)** |  |  | <0.001^2^ |
| All nodes examined are negative | 225 (82.7%) | 2054 (42.5%) |  |
| Any positive nodes | 47 (17.3%) | 2776 (57.5%) |  |
|  | | | |
| **Treatment Characteristics** | | | |
| Treatment Started, Days from Diagnosis: Mean (SD) | 18.0 (38.64) | 27.4 (39.12) | <0.001^1^ |
| First Surgical Procedure, Days from Diagnosis: Mean (SD) | 16.8 (33.30) | 25.9 (42.73) | <0.001^1^ |
|  | | | |
| **Surgery, n (%)** |  |  | <0.001^2^ |
| No | 12 (3.1%) | 1999 (27.5%) |  |
| Yes | 372 (96.9%) | 5277 (72.5%) |  |
|  | | | |
| **Surgical Margin Status, n (%)** |  |  | <0.001^2^ |
| No residual tumor, all margins negative | 310 (85.6%) | 3099 (63.2%) |  |
| Residual tumor, not otherwise specified | 19 (5.2%) | 743 (15.2%) |  |
| Microscopic residual tumor | 33 (9.1%) | 975 (19.9%) |  |
| Macroscopic residual tumor | 0 (0.0%) | 85 (1.7%) |  |
|  | | | |
| **Radiation Therapy, n (%)** |  |  | <0.001^2^ |
| No | 261 (69.4%) | 2512 (34.5%) |  |
| Yes | 123 (30.6%) | 4764 (65.5%) |  |
|  | | | |
| **Chemotherapy, n (%)** |  |  | <0.001^2^ |
| No | 368 (95.8%) | 5951 (81.8%) |  |
| Yes | 16 (4.2%) | 1325 (18.2%) |  |
|  | | | |
| **Clinical Grades and AJCC Pathologic Staging** | | | |
| **Grade, n (%)** |  |  | <0.001^2^ |
| Well differentiated, moderately differentiated | 107 (93.9%) | 1870 (40.1%) |  |
| Poorly differentiated | 4 (3.5%) | 2698 (57.8%) |  |
| Undifferentiated | 3 (2.6%) | 96 (2.1%) |  |
|  | | | |
| **AJCC Pathologic Stage Group, n (%)** |  |  | <0.001^2^ |
| pStage I | 84 (46.9%) | 369 (9.8%) |  |
| pStage II | 48 (26.8%) | 580 (15.4%) |  |
| pStage III | 29 (16.2%) | 1054 (28.0%) |  |
| pStage IV | 0 (0.0%) | 46 (1.2%) |  |
| pStage IVA | 14 (7.8%) | 1455 (38.6%) |  |
| pStage IVB | 1 (0.6%) | 158 (4.2%) |  |
| pStage IVC | 3 (1.7%) | 104 (2.8%) |  |
| ^1^Wilcoxon Rank Sum p-value; ^2^Fisher Exact p-value | | | |

Supplemental Table 3: Demographic, clinical, treatment, clinical grade, and AJCC pathologic staging characteristics of MASC vs. Adenocarcinoma.

|  | **Histological Type** | |  |
| --- | --- | --- | --- |
|  | **MASC (N=384)** | **Adenocarcinoma (N=4029)** | **P-value** |
| **Demographic Characteristics** | | | |
| Age at Diagnosis, Mean (SD) | 53.4 (17.17) | 66.1 (13.90) | <0.001^1^ |
| Sex: Male, n (%) | 195 (50.8%) | 2584 (64.1%) | <0.001^2^ |
|  | | | |
| **Race, n (%)** |  |  | <0.001^2^ |
| White | 298 (77.6%) | 3414 (84.9%) |  |
| Black | 39 (10.2%) | 397 (9.9%) |  |
| Asian/Pacific Islander | 35 (9.1%) | 112 (2.8%) |  |
| Other/Unknown | 12 (3.1%) | 96 (2.4%) |  |
|  | | | |
| **Spanish Hispanic Origin, n (%)** |  |  | <0.001^2^ |
| Non-Spanish, non-Hispanic | 349 (90.9%) | 3622 (89.9%) |  |
| Spanish, Hispanic | 29 (7.6%) | 197 (4.9%) |  |
| Unknown | 6 (1.6%) | 210 (5.2%) |  |
|  | | | |
| **Clinical Characteristics** | | | |
| Lympho-Vascular Invasion: Not present, n (%) | 294 (92.2%) | 948 (59.9%) | <0.001^2^ |
|  | | | |
| **Primary Site, n (%)** |  |  | <0.001^2^ |
| Parotid gland | 326 (84.9%) | 3004 (74.6%) |  |
| Submandibular gland | 31 (8.1%) | 477 (11.8%) |  |
| Sublingual gland | 0 (0.0%) | 34 (0.8%) |  |
| Overlapping lesion of major salivary glands | 3 (0.8%) | 9 (0.2%) |  |
| Major salivary gland, not otherwise specified | 24 (6.3%) | 505 (12.5%) |  |
|  | | | |
| **Regional Lymph Nodes Positive, n (%)** |  |  | <0.001^2^ |
| All nodes examined are negative | 225 (82.7%) | 1028 (39.6%) |  |
| Any positive nodes | 47 (17.3%) | 3001 (60.4%) |  |
|  | | | |
| **Treatment Characteristics** | | | |
| Treatment Started, Days from Diagnosis: Mean (SD) | 18.0 (38.64) | 24.9 (34.85) | <0.001^1^ |
| First Surgical Procedure, Days from Diagnosis: Mean (SD) | 16.8 (33.30) | 22.8 (36.27) | <0.001^1^ |
|  | | | |
| **Surgery, n (%)** |  |  | <0.001^2^ |
| No | 12 (3.1%) | 937 (23.3%) |  |
| Yes | 372 (96.9%) | 3092 (76.6%) |  |
|  | | | |
| **Surgical Margin Status, n (%)** |  |  | <0.001^2^ |
| No residual tumor, all margins negative | 310 (85.6%) | 1802 (62.6%) |  |
| Residual tumor, not otherwise specified | 19 (5.2%) | 429 (14.9%) |  |
| Microscopic residual tumor | 33 (9.1%) | 605 (21.0%) |  |
| Macroscopic residual tumor | 0 (0.0%) | 43 (1.5%) |  |
|  | | | |
| **Radiation Therapy, n (%)** |  |  | <0.001^2^ |
| No | 261 (69.4%) | 1369 (35.2%) |  |
| Yes | 123 (30.6%) | 2660 (64.8%) |  |
|  | | | |
| **Chemotherapy, n (%)** |  |  | <0.001^2^ |
| No | 368 (95.8%) | 2704 (81.8%) |  |
| Yes | 16 (4.2%) | 1325 (18.2%) |  |
|  | | | |
| **Clinical Grades and AJCC Pathologic Staging** | | | |
| **Grade, n (%)** |  |  | <0.001^2^ |
| Well differentiated, moderately differentiated | 107 (93.9%) | 851 (31.4%) |  |
| Poorly differentiated | 4 (3.5%) | 1591 (58.6%) |  |
| Undifferentiated | 3 (2.6%) | 271 (10.0%) |  |
|  | | | |
| **AJCC Pathologic Stage Group, n (%)** |  |  | <0.001^2^ |
| pStage I | 84 (46.9%) | 427 (17.9%) |  |
| pStage II | 48 (26.8%) | 318 (13.3%) |  |
| pStage III | 29 (16.2%) | 403 (16.9%) |  |
| pStage IV | 0 (0.0%) | 43 (1.8%) |  |
| pStage IVA | 14 (7.8%) | 932 (39.0%) |  |
| pStage IVB | 1 (0.6%) | 74 (3.1%) |  |
| pStage IVC | 3 (1.7%) | 192 (8.0%) |  |
| ^1^Wilcoxon Rank Sum p-value; ^2^Fisher Exact p-value | | | |

Supplemental Table 4: Demographic, clinical, treatment, clinical grade, and AJCC pathologic staging characteristics of MASC vs. Adenoid Cystic Carcinoma (ACC).

|  | **Histological Type** | |  |
| --- | --- | --- | --- |
|  | **MASC (N=384)** | **ACC (N=4988)** | **P-value** |
| **Demographic Characteristics** | | | |
| Age at Diagnosis, Mean (SD) | 53.4 (17.17) | 58.4 (15.75) | <0.001^1^ |
| Sex: Male, n (%) | 195 (50.8%) | 2001 (40.1%) | <0.001^2^ |
|  | | | |
| **Race, n (%)** |  |  | 0.0664^2^ |
| White | 298 (77.6%) | 4003 (80.5%) |  |
| Black | 39 (10.2%) | 529 (10.6%) |  |
| Asian/Pacific Islander | 35 (9.1%) | 286 (5.8%) |  |
| Other/Unknown | 12 (3.1%) | 154 (3.1%) |  |
|  | | | |
| **Spanish Hispanic Origin, n (%)** |  |  | 0.0076^2^ |
| Non-Spanish, non-Hispanic | 349 (90.9%) | 4405 (88.3%) |  |
| Spanish, Hispanic | 29 (7.6%) | 331 (6.6%) |  |
| Unknown | 6 (1.6%) | 252 (5.1%) |  |
|  | | | |
| **Clinical Characteristics** | | | |
| Lympho-Vascular Invasion: Not present, n (%) | 294 (92.2%) | 1926 (79.4%) | <0.001^2^ |
|  | | | |
| **Primary Site, n (%)** |  |  | <0.001^2^ |
| Parotid gland | 326 (84.9%) | 2427 (48.7%) |  |
| Submandibular gland | 31 (8.1%) | 1915 (38.4%) |  |
| Sublingual gland | 0 (0.0%) | 206 (4.1%) |  |
| Overlapping lesion of major salivary glands | 3 (0.8%) | 23 (0.5%) |  |
| Major salivary gland, not otherwise specified | 24 (6.3%) | 417 (8.4%) |  |
|  | | | |
| **Regional Lymph Nodes Positive, n (%)** |  |  | 0.0606^2^ |
| All nodes examined are negative | 225 (82.7%) | 2401 (77.5%) |  |
| Any positive nodes | 47 (17.3%) | 697 (22.5%) |  |
|  | | | |
| **Treatment Characteristics** | | | |
| Treatment Started, Days from Diagnosis: Mean (SD) | 18.0 (38.64) | 20.6 (44.47) | 0.0026^1^ |
| First Surgical Procedure, Days from Diagnosis: Mean (SD) | 16.8 (33.30) | 18.5 (41.78) | 0.0373^1^ |
|  | | | |
| **Surgery, n (%)** |  |  | <0.001^2^ |
| No | 12 (3.1%) | 552 (11.1%) |  |
| Yes | 372 (96.9%) | 4436 (88.9%) |  |
|  | | | |
| **Surgical Margin Status, n (%)** |  |  | <0.001^2^ |
| No residual tumor, all margins negative | 310 (85.6%) | 2163 (51.1%) |  |
| Residual tumor, not otherwise specified | 19 (5.2%) | 771 (18.2%) |  |
| Microscopic residual tumor | 33 (9.1%) | 1219 (28.8%) |  |
| Macroscopic residual tumor | 0 (0.0%) | 77 (1.8%) |  |
|  | | | |
| **Radiation Therapy, n (%)** |  |  | <0.001^2^ |
| No | 261 (69.4%) | 1373 (28.5%) |  |
| Yes | 123 (30.6%) | 3615 (71.5%) |  |
|  | | | |
| **Chemotherapy, n (%)** |  |  | <0.001^2^ |
| No | 368 (95.8%) | 4479 (89.8%) |  |
| Yes | 16 (4.2%) | 509 (10.2%) |  |
|  | | | |
| **Clinical Grades and AJCC Pathologic Staging** | | | |
| **Grade, n (%)** |  |  | <0.001^2^ |
| Well differentiated, moderately differentiated | 107 (93.9%) | 1340 (69.7%) |  |
| Poorly differentiated | 4 (3.5%) | 441 (23.0%) |  |
| Undifferentiated | 3 (2.6%) | 140 (7.3%) |  |
|  | | | |
| **AJCC Pathologic Stage Group, n (%)** |  |  | <0.001^2^ |
| pStage I | 84 (46.9%) | 773 (24.4%) |  |
| pStage II | 48 (26.8%) | 716 (22.6%) |  |
| pStage III | 29 (16.2%) | 758 (24.0%) |  |
| pStage IV | 0 (0.0%) | 15 (0.5%) |  |
| pStage IVA | 14 (7.8%) | 606 (19.2%) |  |
| pStage IVB | 1 (0.6%) | 106 (3.4%) |  |
| pStage IVC | 3 (1.7%) | 189 (6.0%) |  |
| ^1^Wilcoxon Rank Sum p-value; ^2^Fisher Exact p-value | | | |

Supplemental Table 5: Demographic, clinical, treatment, clinical grade, and AJCC pathologic staging characteristics of MASC vs. Mucoepidermoid Carcinoma.

|  | **Histological Type** | |  |
| --- | --- | --- | --- |
|  | **MASC (N=384)** | **Mucoepidermoid Carcinoma (N=9836)** | **P-value** |
| **Demographic Characteristics** | | | |
| Age at Diagnosis, Mean (SD) | 53.4 (17.17) | 57.2 (17.15) | <0.001^1^ |
| Sex: Male, n (%) | 195 (50.8%) | 4489 (45.6%) | 0.0472^2^ |
|  | | | |
| **Race, n (%)** |  |  | <0.001^2^ |
| White | 298 (77.6%) | 7475 (76.2%) |  |
| Black | 39 (10.2%) | 1570 (16.0%) |  |
| Asian/Pacific Islander | 35 (9.1%) | 465 (4.7%) |  |
| Other/Unknown | 12 (3.1%) | 297 (3.0%) |  |
|  | | | |
| **Spanish Hispanic Origin, n (%)** |  |  | 0.0163^2^ |
| Non-Spanish, non-Hispanic | 349 (90.9%) | 8659 (88.0%) |  |
| Spanish, Hispanic | 29 (7.6%) | 717 (7.3%) |  |
| Unknown | 6 (1.6%) | 460 (4.7%) |  |
|  | | | |
| **Clinical Characteristics** | | | |
| Lympho-Vascular Invasion: Not present, n (%) | 294 (92.2%) | 4685 (88.5%) | 0.0429^2^ |
|  | | | |
| **Primary Site, n (%)** |  |  | <0.001^2^ |
| Parotid gland | 326 (84.9%) | 8403 (85.4%) |  |
| Submandibular gland | 31 (8.1%) | 866 (8.8%) |  |
| Sublingual gland | 0 (0.0%) | 175 (1.8%) |  |
| Overlapping lesion of major salivary glands | 3 (0.8%) | 10 (0.1%) |  |
| Major salivary gland, not otherwise specified | 24 (6.3%) | 382 (3.9%) |  |
|  | | | |
| **Regional Lymph Nodes Positive, n (%)** |  |  | 0.0373^2^ |
| All nodes examined are negative | 225 (82.7%) | 5064 (75.9%) |  |
| Any positive nodes | 47 (17.3%) | 1612 (24.1%) |  |
|  | | | |
| **Treatment Characteristics** | | | |
| Treatment Started, Days from Diagnosis: Mean (SD) | 18.0 (38.64) | 15.9 (32.58) | 0.5948^1^ |
| First Surgical Procedure, Days from Diagnosis: Mean (SD) | 16.8 (33.30) | 15.3 (32.86) | 0.9185^1^ |
|  | | | |
| **Surgery, n (%)** |  |  | <0.001^2^ |
| No | 12 (3.1%) | 666 (6.8%) |  |
| Yes | 372 (96.9%) | 9170 (93.2%) |  |
|  | | | |
| **Surgical Margin Status, n (%)** |  |  | <0.001^2^ |
| No residual tumor, all margins negative | 310 (85.6%) | 6634 (74.9%) |  |
| Residual tumor, not otherwise specified | 19 (5.2%) | 829 (9.4%) |  |
| Microscopic residual tumor | 33 (9.1%) | 1320 (14.9%) |  |
| Macroscopic residual tumor | 0 (0.0%) | 77 (0.9%) |  |
|  | | | |
| **Radiation Therapy, n (%)** |  |  | <0.001^2^ |
| No | 261 (69.4%) | 5329 (55.7%) |  |
| Yes | 123 (30.6%) | 4507 (44.3%) |  |
|  | | | |
| **Chemotherapy, n (%)** |  |  | <0.001^2^ |
| No | 368 (95.8%) | 9125 (92.8%) |  |
| Yes | 16 (4.2%) | 711 (7.2%) |  |
|  | | | |
| **Clinical Grades and AJCC Pathologic Staging** | | | |
| **Grade, n (%)** |  |  | <0.001^2^ |
| Well differentiated, moderately differentiated | 107 (93.9%) | 5333 (72.6%) |  |
| Poorly differentiated | 4 (3.5%) | 1412 (19.2%) |  |
| Undifferentiated | 3 (2.6%) | 602 (8.2%) |  |
|  | | | |
| **AJCC Pathologic Stage Group, n (%)** |  |  | 0.0675^2^ |
| pStage I | 84 (46.9%) | 2875 (45.2%) |  |
| pStage II | 48 (26.8%) | 1309 (20.6%) |  |
| pStage III | 29 (16.2%) | 999 (15.7%) |  |
| pStage IV | 0 (0.0%) | 30 (0.5%) |  |
| pStage IVA | 14 (7.8%) | 983 (15.4%) |  |
| pStage IVB | 1 (0.6%) | 82 (1.3%) |  |
| pStage IVC | 3 (1.7%) | 87 (1.4%) |  |
| ^1^Wilcoxon Rank Sum p-value; ^2^Fisher Exact p-value | | | |

Supplemental Table 6: Demographic, clinical, treatment, clinical grade, and AJCC pathologic staging characteristics of MASC vs. Polymorphous Adenocarcinoma.

|  | **Histological Type** | |  |
| --- | --- | --- | --- |
|  | **MASC (N=384)** | **Polymorphous Adenocarcinoma (N=168)** | **P-value** |
| **Demographic Characteristics** | | | |
| Age at Diagnosis, Mean (SD) | 53.4 (17.17) | 62.3 (14.26) | <0.001^1^ |
| Sex: Male, n (%) | 195 (50.8%) | 68 (40.5%) | 0.0257^2^ |
|  | | | |
| **Race, n (%)** |  |  | 0.0048^2^ |
| White | 298 (77.6%) | 125 (74.4%) |  |
| Black | 39 (10.2%) | 31 (18.5%) |  |
| Asian/Pacific Islander | 35 (9.1%) | 5 (3.0%) |  |
| Other/Unknown | 12 (3.1%) | 7 (4.2%) |  |
|  | | | |
| **Spanish Hispanic Origin, n (%)** |  |  | 0.0060^2^ |
| Non-Spanish, non-Hispanic | 349 (90.9%) | 142 (84.5%) |  |
| Spanish, Hispanic | 29 (7.6%) | 15 (8.9%) |  |
| Unknown | 6 (1.6%) | 11 (6.5%) |  |
|  | | | |
| **Clinical Characteristics** | | | |
| Lympho-Vascular Invasion: Not present, n (%) | 294 (92.2%) | 63 (87.5%) | 0.2047^2^ |
|  | | | |
| **Primary Site, n (%)** |  |  | <0.001^2^ |
| Parotid gland | 326 (84.9%) | 58 (34.5%) |  |
| Submandibular gland | 31 (8.1%) | 23 (13.7%) |  |
| Sublingual gland | 0 (0.0%) | 11 (6.5%) |  |
| Overlapping lesion of major salivary glands | 3 (0.8%) | 2 (1.2%) |  |
| Major salivary gland, not otherwise specified | 24 (6.3%) | 74 (44.0%) |  |
|  | | | |
| **Regional Lymph Nodes Positive, n (%)** |  |  | 0.8163^2^ |
| All nodes examined are negative | 225 (82.7%) | 41 (85.4%) |  |
| Any positive nodes | 47 (17.3%) | 7 (14.6%) |  |
|  | | | |
| **Treatment Characteristics** | | | |
| Treatment Started, Days from Diagnosis: Mean (SD) | 18.0 (38.64) | 21.5 (42.43) | 0.1976^1^ |
| First Surgical Procedure, Days from Diagnosis: Mean (SD) | 16.8 (33.30) | 20.7 (42.47) | 0.9185^1^ |
|  | | | |
| **Surgery, n (%)** |  |  | 0.7616^1^ |
| No | 12 (3.1%) | 21 (12.5%) |  |
| Yes | 372 (96.9%) | 147 (87.5%) |  |
|  | | | |
| **Surgical Margin Status, n (%)** |  |  | <0.001^2^ |
| No residual tumor, all margins negative | 310 (85.6%) | 96 (70.1%) |  |
| Residual tumor, not otherwise specified | 19 (5.2%) | 18 (13.1%) |  |
| Microscopic residual tumor | 33 (9.1%) | 23 (16.8%) |  |
| Macroscopic residual tumor | 0 (0.0%) | 0 (0.0%) |  |
|  | | | |
| **Radiation Therapy, n (%)** |  |  | 0.6241^2^ |
| No | 261 (69.4%) | 122 (72.6%) |  |
| Yes | 123 (30.6%) | 46 (27.4%) |  |
|  | | | |
| **Chemotherapy, n (%)** |  |  | 0.2447^2^ |
| No | 368 (95.8%) | 158 (94.0%) |  |
| Yes | 16 (4.2%) | 10 (6.0%) |  |
|  | | | |
| **Clinical Grades and AJCC Pathologic Staging** | | | |
| **Grade, n (%)** |  |  | 0.2246^2^ |
| Well differentiated, moderately differentiated | 107 (93.9%) | 116 (97.5%) |  |
| Poorly differentiated | 4 (3.5%) | 2 (1.7%) |  |
| Undifferentiated | 3 (2.6%) | 1 (0.8%) |  |
|  | | | |
| **AJCC Pathologic Stage Group, n (%)** |  |  | 0.3394^2^ |
| pStage I | 84 (46.9%) | 40 (47.1%) |  |
| pStage II | 48 (26.8%) | 25 (29.4%) |  |
| pStage III | 29 (16.2%) | 11 (12.9%) |  |
| pStage IV | 0 (0.0%) | 1 (1.2%) |  |
| pStage IVA | 14 (7.8%) | 3 (3.5%) |  |
| pStage IVB | 1 (0.6%) | 2 (2.4%) |  |
| pStage IVC | 3 (1.7%) | 3 (3.5%) |  |
| ^1^Wilcoxon Rank Sum p-value; ^2^Fisher Exact p-value | | | |

Supplemental Table 7: Demographic, clinical, treatment, clinical grade, and AJCC pathologic staging characteristics of MASC vs. Acinic Cell Carcinoma.

|  | **Histological Type** | |  |
| --- | --- | --- | --- |
|  | **MASC (N=384)** | **Acinic Cell Carcinoma (N=5103)** | **P-value** |
| **Demographic Characteristics** | | | |
| Age at Diagnosis, Mean (SD) | 53.4 (17.17) | 54.6 (16.97) | 0.1598^1^ |
| Sex: Male, n (%) | 195 (50.8%) | 1981 (38.8%) | <0.001^2^ |
|  | | | |
| **Race, n (%)** |  |  | <0.001^2^ |
| White | 298 (77.6%) | 4330 (85.1%) |  |
| Black | 39 (10.2%) | 477 (9.4%) |  |
| Asian/Pacific Islander | 35 (9.1%) | 143 (2.8%) |  |
| Other/Unknown | 12 (3.1%) | 140 (2.8%) |  |
|  | | | |
| **Spanish Hispanic Origin, n (%)** |  |  | 0.0078^2^ |
| Non-Spanish, non-Hispanic | 349 (90.9%) | 4522 (88.6%) |  |
| Spanish, Hispanic | 29 (7.6%) | 327 (6.4%) |  |
| Unknown | 6 (1.6%) | 254 (5.0%) |  |
|  | | | |
| **Clinical Characteristics** | | | |
| Lympho-Vascular Invasion: Not present, n (%) | 294 (92.2%) | 2348 (85.6%) | <0.001^2^ |
|  | | | |
| **Primary Site, n (%)** |  |  | <0.001^2^ |
| Parotid gland | 326 (84.9%) | 4928 (96.6%) |  |
| Submandibular gland | 31 (8.1%) | 72 (1.4%) |  |
| Sublingual gland | 0 (0.0%) | 4 (0.1%) |  |
| Overlapping lesion of major salivary glands | 3 (0.8%) | 5 (0.1%) |  |
| Major salivary gland, not otherwise specified | 24 (6.3%) | 94 (1.8%) |  |
|  | | | |
| **Regional Lymph Nodes Positive, n (%)** |  |  | 0.0236^2^ |
| All nodes examined are negative | 225 (82.7%) | 3007 (85.7%) |  |
| Any positive nodes | 47 (17.3%) | 501 (14.3%) |  |
|  | | | |
| **Treatment Characteristics** | | | |
| Treatment Started, Days from Diagnosis: Mean (SD) | 18.0 (38.64) | 17.4 (35.68) | 0.2488^1^ |
| First Surgical Procedure, Days from Diagnosis: Mean (SD) | 16.8 (33.30) | 17.0 (35.34) | 0.3097^1^ |
|  | | | |
| **Surgery, n (%)** |  |  | 0.7616^1^ |
| No | 12 (3.1%) | 4847 (95.0%) |  |
| Yes | 372 (96.9%) | 256 (5.0%) |  |
|  | | | |
| **Surgical Margin Status, n (%)** |  |  | <0.001^2^ |
| No residual tumor, all margins negative | 310 (85.6%) | 3359 (72.6%) |  |
| Residual tumor, not otherwise specified | 19 (5.2%) | 482 (10.4%) |  |
| Microscopic residual tumor | 33 (9.1%) | 752 (16.3%) |  |
| Macroscopic residual tumor | 0 (0.0%) | 34 (0.7%) |  |
|  | | | |
| **Radiation Therapy, n (%)** |  |  | <0.001^2^ |
| No | 261 (69.4%) | 2856 (56.0%) |  |
| Yes | 123 (30.6%) | 2247 (44.0%) |  |
|  | | | |
| **Chemotherapy, n (%)** |  |  | 0.5442^2^ |
| No | 368 (95.8%) | 4846 (95.0%) |  |
| Yes | 16 (4.2%) | 257 (5.0%) |  |
|  | | | |
| **Clinical Grades and AJCC Pathologic Staging** | | | |
| **Grade, n (%)** |  |  | 0.0046^2^ |
| Well differentiated, moderately differentiated | 107 (93.9%) | 1702 (85.0%) |  |
| Poorly differentiated | 4 (3.5%) | 217 (10.8%) |  |
| Undifferentiated | 3 (2.6%) | 84 (4.2%) |  |
|  | | | |
| **AJCC Pathologic Stage Group, n (%)** |  |  | 0.5001^2^ |
| pStage I | 84 (46.9%) | 1364 (40.2%) |  |
| pStage II | 48 (26.8%) | 1135 (33.5%) |  |
| pStage III | 29 (16.2%) | 513 (15.1%) |  |
| pStage IV | 0 (0.0%) | 1 (0.0%) |  |
| pStage IVA | 14 (7.8%) | 8 (0.2%) |  |
| pStage IVB | 1 (0.6%) | 300 (8.8%) |  |
| pStage IVC | 3 (1.7%) | 37 (1.1%) |  |
| ^1^Wilcoxon Rank Sum p-value; ^2^Fisher Exact p-value | | | |
